# The Relationship between Risk Perception of Cell Phones and Objective Knowledge of EMF in Korea

**DOI:** 10.3390/ijerph17197207

**Published:** 2020-10-01

**Authors:** Myung-Soon Seo, Jae-Wook Choi, Kyung-Hee Kim, Hyung-Do Choi

**Affiliations:** 1 Department of Public Health, College of Medicine, Korea University, Seoul 02481, Korea; lundy3972@gmail.com; 2 Institute for Environmental Health, Korea University, Seoul 02481, Korea; kyonghee80@korea.ac.kr; 3 Department of Preventive Medicine, College of Medicine, Korea University, Seoul 02481, Korea; 4 Radio and Satellite Division, Electronics and Telecommunications Research Institute, Daejeon 34129, Korea; choihd@etri.re.kr

**Keywords:** risk awareness, mobile phone, knowledge level of RF-EMF, uncontrollability, hierarchical multiple regression analysis, risk cognition map

## Abstract

This study examines differences between the level of objective knowledge regarding radio-frequency electromagnetic fields (RF-EMF) and risk perception of cell phones in Korea. We also investigate the extent to which socio-demographic factors, perceived EMF exposure, objective knowledge regarding EMF, and psychological factors influence the risk perception of cell phones using hierarchical multiple regression. All 3393 study subjects completed a survey measuring the degree of risk perception of EMF. They were sampled in accordance with representative proportions of sex, age group, and region of residence as shown in the 2019 Resident Registration Population Statistics reported by Korea. The variables that have the most influence on risk perception of cell phones can be induced from the beta values for each variable: The subjective factor, perceived level of exposure to EMF (β = 0.253), was more strongly related to risk perception of cell phones than level of knowledge regarding EMF, an objective factor in this study. Of the psychological factors, Dreadfulness (β = 0.331), Personal knowledge (β = −174), and Familiarity (β = −089) influenced risk perceptions of cell phones; Controllability did not. On the risk cognition map, people though that it was easy to control risk related to Cell phone use in daily life, while risk related to High technology was uncontrollable.

## 1. Introduction

South Korea’s cell phone usage rate was 95%, the highest among 27 developed countries, in 2019 [1]. An examination of the changes in usage rates by age group between 2015 and 2018 found that 18–34 year olds showed a change of 99–100%, while for those 50 years old and older, cell phone use safety is high. Cell phones use radio frequency electromagnetic fields (RF-EMF), and many studies have been conducted on the health effects of EMFs such as RF: Generalized EMF Research using Novel Methods (GERoNiMO, ISGlobal Radiation Programmes), Cohort Study of Mobile Phone Use and Health (COSMOS), Radio Frequency Electromagnetic Fields Exposure and Brain Development (REMBRANDT), and the Study on Communication Technology, Environment and Brain Tumors in Young People (Mobi-Kids Studies) [2,3,4,5]. The International Agency for Research on Cancer (IARC) has classified radiofrequency electromagnetic fields as Group 2B, possibly carcinogenic, for all types of radiation within the radiofrequency part of the electromagnetic spectrum, including the radiation emitted by base-station antennas, radio/TV towers, radar, Wi-Fi, smart meters, etc. [6].

Research has been carried out on the awareness and risk perception of EMF. Non-professional perceptions of the risk of RF-EMF exposure are not scientific facts, but rather are decisively influenced by intuitive beliefs [7]. Zwick [8] proposed measuring the subjective importance of various risks and conducted a comparative evaluation of various risks in technology, society, environment, and daily life in a risk awareness questionnaire. Recently, research on risk perceptions of RF-EMF has led to concerns in everyday life, that is, the degree to which people think about or discuss risk issues [9]. On the other hand, the results of research on the impact of knowledge related to EMF are as follows: Kennedy et al. [10] and MacGregor et al. [11] found an increased risk perception by people with a high degree of knowledge. Frederik et al. [12] found that better exposure to knowledge led to higher risk perception, although the effect strength was rather small and there were significant differences in knowledge for mobile phone risk perception. However, other studies have shown risk perception to be moderately negatively correlated with knowledge [13,14]. For mobile communication, it was found that the general public had only limited knowledge [15,16]. Overall, there is mixed evidence regarding the association between risk perception and knowledge [17]. For this reason, this study attempts to grasp the relevance of knowledge in Koreans’ risk perception with respect to electromagnetic fields.

In Korea, research on the risk perception of EMF has generally been focused on establishing laws and regulations necessary for EMF measurement and risk management to develop appropriate guidelines; only a few studies of risk perception and EMF have been concerned with personal perception instead of objective knowledge [18,19,20].

There have been some studies of subjective knowledge with respect to the risk perception of EMF, but this study is focused on the differences in risk perception of RF-EMF by measuring objective knowledge. In this study, with respect to the level of knowledge, the factors for knowledge were classified into three categories: the level of education (officially recorded), the level of subjective knowledge of electromagnetic fields, and the level of objective knowledge of electromagnetic fields. In particular, this study is the first to measure Koreans’ objective knowledge of electromagnetic fields. In addition to the objective knowledge level, the questionnaire also included questions about how much electromagnetic radiation people were exposed to in daily life and subjective knowledge levels regarding the risk perception of cell phones. We examined the extent to which each of five psychological factors influences the risk perception of cell phones: Personal knowledge, Controllability, Dreadfulness, Risk known to science, and Familiarity. In this way, as the perception of risk factors varies depending on personal experience and psychological factors, we presented risk cognitive maps, which average the risk perception of individuals for each risk factor in this study.

## 2. Materials and Methods

### 2.1. Research Questions 

The research questions of this study are:

RQ 1) What are the factors influencing the risk perception of cell phones?
RQ 2) Does the level of knowledge of EMF affect the risk perception of cell phones?
RQ 3) How much do socio-demographic characteristics, psychological factors, perceived level of exposures to EMF, and objective knowledge regarding EMF affect the risk perception of cell phones?

**Figure 1:**
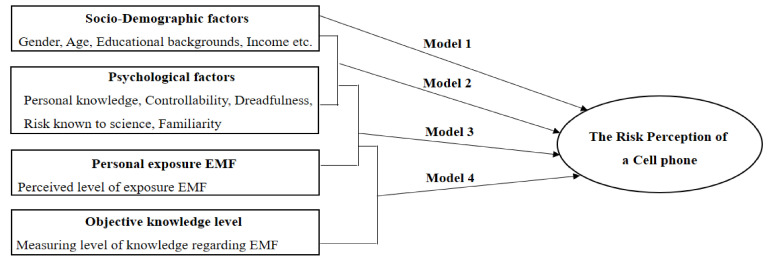
Conceptual model of research on risk perceptions of cell phones. EMF: Electromagnetic Fields.

Slovic [21,22] proposed that risk perception is essentially subjective and Dunwoody and Neuwirth [23] grouped risk characteristics from the psychometric paradigm along two dimensions, cognitive and emotional. In this study, we selected Personal knowledge, Controllability, and Familiarity along the cognitive dimension, and Dreadfulness along the emotional dimension. Through a review of the above literature, we constructed the research model shown in Figure 1 to determine the relevance and effects of objective factors and other factors on the risk perception of cell phones. 

According to Slovic [21,22], risk perception is determined by psychological and subjective factors. It is known that subjective influences (gender, age, socio-economic level, etc.) have a great influence on perception in the existing electromagnetic wave risk recognition studies [7,8]. For these reasons, through hierarchical regression analysis, variables related to demographic factors, psychological factors, and subjective factors (subjectively felt exposure to electromagnetic fields, subjective knowledge level of electromagnetic fields) were sequentially introduced in the risk perception of electromagnetic fields. Objective factors to be reported (level of objective knowledge of electromagnetic fields measured in this study) were added last. Through this stepwise analysis, the magnitude of the relative influence of the independent variable was identified in order.

### 2.2. Patient and Public Recruitment

**Figure 2:**
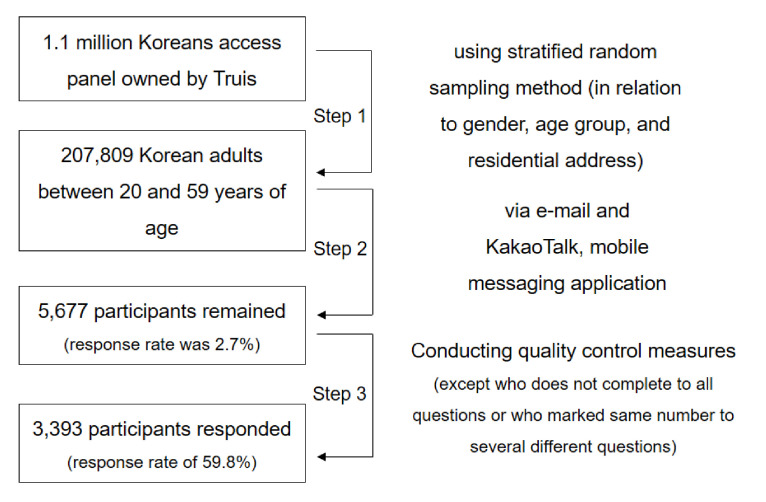
The recruitment process of patient and public in this study.

A market research company in Korea, Truis, has access to a panel of 1.1 million Koreans, and 207,809 Korean adults were selected by gender, age group and residential area using random sampling. They received an e-mail and a Kakao Talk message inviting them to participate in the survey. The initial response rate was 2.7%; 5677 responded to the survey. After conducting quality control measures (excluding those who did not complete all questions or who had marked the same number for several different questions), there were 3393 final participants, excluding respondents who did not complete the survey. All quality control of data for this survey was ensured by the Truis company. This process described above is shown as a diagram (Figure 2).

### 2.3. Study Subjects in Survey

In South Korea, 3393 study subjects completed a survey designed to measure the degree of risk perception of EMF. Study subjects were between the ages of 20 and 59 and resided in 13 regions: the city of Seoul, Busan, Incheon, Daegu, Daejeon, Gwangju, Ulsan, Kyunggido, Kangwondo, Kyungsangdo, Choongcheongdo (including Sejongsi), Jeonlado, and Jeju Island. They were sampled in accordance with representative proportions of sex, age group, and region of residence as shown in the 2019 Resident Registration Population Statistics reported by the Ministry of Security and Public Administration of Korea.

### 2.4. Questionnaire

The survey was conducted to investigate the degree of risk perception of EMF in South Korea. The questionnaire had five sections: screening questions (SQ), questions on EMF sources: cell phones and microwave ovens (Section A), questions testing objective knowledge of EMF (Section B), questions assessing risk perception in psychometric diagrams (Section C), and demographic questions (DQ). The participants responded to the aforementioned questionnaires by accessing a web-based survey program. Questionnaires were then screened to select relevant variables for our analysis after cleansing to improve the quality of data. In Section A, the subjects responded on a 10-point Likert scale ranging from 1 (low perceived exposure to EMF waves) to 10 (very high exposure), with high knowledge groups (8–10 points), middle knowledge groups (6–7 points), and low knowledge groups (1–5 points). There were a total of 13 questions measuring objective knowledge, and yes/no questions in Section B. Thirty-three levels of Respondents’ knowledge were classified into three groups as follows: upper (7 or more), middle (4 to 6), and lower (3 or less). In Section C, the subjects responded to each question in the psychometric paradigm as follows: Personal knowledge (know nothing = 1, know very well = 10), Controllability (very uncontrollable = 1, very controllable = 10), Dreadfulness (do not feel dread at all = 1, feel great dread = 10), Risk known to science (not known to science = 1, well known to science = 10), and Familiarity (very unfamiliar = 1, very familiar = 10).

### 2.5. Statistical Analysis

Descriptive statistical analyses of demographic characteristics; perceived level of exposure to EMF; level of knowledge regarding EMF; psychological factors (Personal knowledge, Controllability, Dreadfulness, Risk known to science, Familiarity); and risk perception of cell phones were carried out. Risk perception scores of RF-EMF from cell phones were selected as a dependent variable. Hierarchical multiple regression analysis was performed to compare the effects of the independent variables on the risk perception of cell phones. Factor analysis was performed on the perception of risk factors for each electromagnetic wave source based on the psychological paradigm model of P. Slovic. All statistical analyses were performed in SPSS version 22 (IBM, Armonk, NY, USA).

### 2.6. Ethics Statement

This study was conducted after obtaining approval from the Institutional Review Board (IRB) of Korea University (KUIRB-2019-0240-01).

## 3. Results

### 3.1. Socio-Demographic Characteristics

The socio-demographic characteristics of the subjects are shown in Table 1. The study subjects comprised 1760 men (51.9%) and 1633 (48.1%) women over the age of 20. The socio demographic characteristics of male and female respondents were as follows: By age, both men and women were evenly distributed, but those in their 40s were the largest group, with 28.7% and 26.9%, respectively. By region, the three regions of Gyeongki-do, Seoul, and Gyeongsang-do included 53.1% of the male and 54.3% of the female subjects, accounting for more than half of the respondents. By educational background, 53.9% (male) and 50.3% (female) were college graduates, and 48.5% (male) and 45.3% (female) had average monthly household incomes between 3000 and 6000 dollars. 

### 3.2. The Variables Affecting the Risk Perception of Cell Phones

A hierarchical multiple regression analysis was performed to check the effect of objective knowledge level on the risk perception of cell phones while other variables were controlled. The explanatory power of each model can be described by R2 values, such that the explanatory power increases with R2 values: 0.49 for Model 1, 0.297 for Model 2, 0.333 for Model 3, and 0.335 for Model 4. In the analysis of variance, the significance probability of the F-test for each model was 0.000. This is indicated in Table 2. All of the *F*-test significance probabilities were less than 0.05, indicating that each model’s regression model was suitable. In addition, the *p*-values for variance analysis were below 0.001 in all models, indicating suitability for regression analysis. In the hierarchical multiple regression analysis, the variables that have the most influence on risk perception of cell phones can be induced from the beta values for each variable: Dreadfulness (β = 0.331), high group of Perceived level of exposures to EM waves (β = 0.253), Personal knowledge (β = −0.174), middle group of Perceived level of exposures to EM waves (β = 0.113), Familiarity (β = −.089), Female (β = 0.085), 30–39 of Age (β = 0.071), 40–49 of Age (β = 0.066), 50–59 of Age (β = 0.067), and high group of Level of knowledge regarding EMF (β = 0.037), as follows. Among the psychological factors, Dreadfulness was the most influential factor on the risk perception of cell phones, followed by electromagnetic radiation exposure, subjective knowledge level, and objective knowledge level.

### 3.3. The Risk Cognitive Map on Electromagnetic Fields around the Public

**Figure 3:**
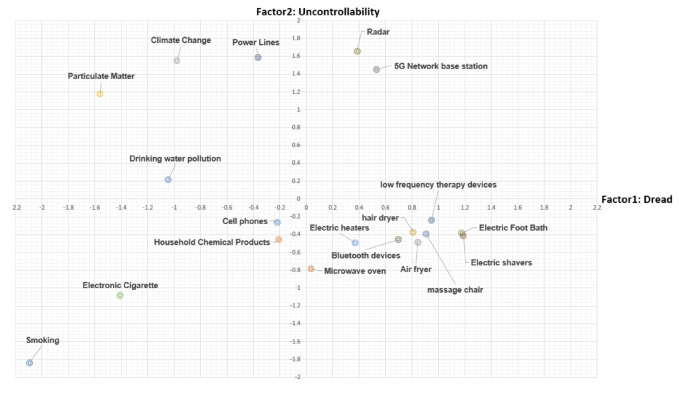
Risk cognitive map on electromagnetic fields around the public.

Factor analysis was performed to reduce the dimensions that affect the personal psychological characteristics for the risk perception of the 19 common risk factors into simpler dimensions, for the purpose of finding the possibility of summarizing eight psychological characteristic variables in two dimensions. In the factor analysis, factors were extracted using the principle component method and the varimax rotation method. Since the perception of risk factors differs according to individual experiences and psychological judgments, we analyzed people’s aspects of risk by using cognitive maps that express individual risk perceptions for each risk factor as an average. Based on P. Slovic’s psychometric paradigm model, a factor analysis was performed on the perception of risk factors for each source of electromagnetic fields, and a risk perception map was created (Figure 3). As a result of factor analysis by electromagnetic wave risk factors, eight variables were divided into two factors. Therefore, the contents extracted as factor 1 were subjective cognition, seriousness to future generation, dreadfulness, Resilience, Risk known to science, Immediacy of the effect of risk, Familiarity, etc., which are called Dread on the X axis, and factor 2 is uncontrollability, Y. Each hazard was marked by naming it uncontrollable. Dread means psychological fear of each risk factor, and uncontrollable means that the individual is an area in which it is difficult to control each risk factor.

As shown in Figure 3, the radar and the 5G Network base station are difficult to control through individual efforts to control risk and are also located in the dread area. It can be seen that dread of new technologies such as 5G base stations is greater, and these are perceived as more dangerous for infrastructure and facilities that are not personally controllable, i.e., where one has no individual control over exposure, such as a THAAD (Terminal High Altitude Area Defense) radar. Power lines, climate change, particulate matter, and drinking water pollution are located in areas with low dread but which are difficult to control by individuals. Cell phones are located in the realm of undread, with household chemical products and electronic cigarette smoking, and can be controlled by the individual. On the other hand, there is dread with respect to household appliances in daily life, such as microwave ovens, electric heaters, hair dryers, and air fryers, but they are located in areas that can be controlled by individual efforts. This suggests that in the case of household appliances, the individual’s ability to select and decide whether or not to use them affects their perception of risk.

## 4. Discussion

This study aimed to investigate factors that affect the risk perception of cell phones. In particular, we focused on the relationship between the level of objective knowledge regarding RF-EMF and the risk perception of cell phones in Korea.

Among demographic factors, gender, age, and household income have been associated with the risk perception of cell phones; the risk perception of women was higher than that of men, corroborating previous studies [18,24]. In addition, the subjective factor, perceived level of exposure to EMF, was more strongly related to the risk perception of cell phones than level of knowledge regarding EMF, which was measured as an objective factor in this study. This finding was confirmed again by the results of hierarchical multiple regression analysis regarding the relationship between psychological factors and risk perception of cell phones. We found that all factors except education level affected the risk perception of cell phones in the results of hierarchical multiple regression analysis. In Model 4, only Dreadfulness (*p* < 0.001, β = 0.331), Personal knowledge (*p* < 0.001, β = 0.174), and Familiarity (*p* < 0.001, β = 0.089), but not Controllability (*p* = 0.548), influenced the risk perception of cell phones among the psychological factors, which confirms the GERoNiMO study results reported in 2018: EMF is invisible and ubiquitous, and exposure to EMF is commonly perceived to be beyond the control of the individual [25]. From this point of view, perceived level of exposure to EMF was related to the risk perception of cell phones, and those who felt that exposure to EMF was high (*p* < 0.001, β = 0.253) showed a greater effect than did those in the middle group (*p* < 0.001, β = 0.113) [26]. However, in objective knowledge, only the group with high knowledge level (*p* < 0.05, β = 0.037) showed effects on the risk perception of cell phones, while the middle group of knowledge level did not. We conclude that the risk perception of cell phones is related to subjective factors, such as the perceived level of exposure to EMF, dreadfulness [21,23], personal knowledge, and familiarity, but not to objective factors, although a high level of knowledge regarding EMF did show a relationship. On the risk cognition map, cell phones are located in area where it can be controllable by the individual’s efforts and also low dread. People might have thought that cell phones’ risks were more controllable, in that an individual can always carry it, rather than other EMF sources.

## 5. Conclusions

We investigated the relationship between objective knowledge and the risk perception of cell phones by measuring objective knowledge using knowledge-related EMF questions in this study drawn from the literature. However, these questions were somewhat limited, in the sense that they referred not only to RF-EMF, but also to other sources of EMF. Likewise, perceived exposure to EMF could be recognized as the total exposure to all electromagnetic fields from ubiquitous devices such as electronic products (microwave ovens, hair driers, etc.) and power lines, as well as cell phones. Despite these limitations, our research has several advantages: We reviewed both subjective and objective factors to determine the relationship between the risk perception of cell phones and level of knowledge regarding EMF in Korea, the nation where the world’s cell phone usage is the highest. As a result of the study, although psychological factors still have the greatest influence on risk perception, subjective knowledge was found to have the greatest influence on Koreans’ risk perception of electromagnetic fields in terms of knowledge and risk perception. The results for the psychological factors, particularly in the cognitive domain, support the findings of the GERoNiMO Project that the risk perception of electromagnetic fields is not controllable [25]. The main results of this study can serve as a scientific basis for various factors that can affect the public’s risk perception of RF-EMF. This can help in communicating with the public and can be expected to play an important role in the risk management of RF-EMF. Furthermore, it can help develop effective communication strategies to reduce risk perception of EMF.

## Figures and Tables

**Table 1:** Socio-demographic characteristics of the study subjects (Male = 1760, Female = 1633).

Variables		Male		Female	
		*n*	%	*n*	%
Age	20–29	391	22.2	371	22.7
	30–39	411	23.4	396	24.2
	40–49	506	28.7	439	26.9
	50–59	452	25.7	427	26.1
Region	Seoul	313	17.8	320	19.6
	Busan	129	7.3	109	6.7
	Incheon	112	6.4	99	6.1
	Daegu	88	5.0	82	5.0
	Daejeon	59	3.4	55	3.4
	Gwangju	59	3.4	54	3.3
	Ulsan	49	2.8	44	2.7
	Kyungkido	438	24.9	399	24.4
	Kangwondo	56	3.2	55	3.4
	Kyungsangdo	183	10.4	169	10.3
	Choongcheongdo (including Sejongsi)	134	7.6	119	7.3
	Jeonlado	112	6.4	103	6.3
	Jeju-Island	28	1.6	25	1.5
Level of education	High school graduate or less	242	13.8	351	21.5
	College graduate	948	53.9	821	50.3
	More than college graduate	570	32.4	461	28.2
Household Income	<3000	490	27.8	475	29.1
	3000≤ & <6000 *	853	48.5	740	45.3
	≥6000	417	23.7	418	25.6

* ≤ &< means more than dollars and less than dollars.

**Table 2:** Result of hierarchical multiple regression analysis of risk perceptions of cell phones.

Variables		Model 1			Model 2			Model 3			Model 4			
		β	*t*	*p*-Value	β	*t*	*p*-Value	β	*t*	*p*-Value	β	*t*	*p*-Value	Tolerance
Gender	Male (ref.)	−	−	−	−	−	−	−	−	−	−	−	−	−
	Female	0.195	11.530	<0.001	0.110	7.461	<0.001	0.081	5.542	<0.001	0.085	5.787	<0.001	1.090
Age	20–29 (ref.)	−	−	−	−	−	−	−	−	−	−	−	−	−
	30–39	0.094	4.463	<0.001	0.066	3.622	<0.001	0.074	4.133	<0.001	0.071	4.010	<0.001	1.610
	40–49	0.081	3.730	<0.001	0.039	2.103	0.036 *	0.068	3.686	<0.001	0.066	3.571	<0.001	1.718
	50–59	0.079	3.630	<0.001	0.028	1.519	0.129	0.068	3.668	<0.001	0.067	3.622	<0.001	1.746
Level of Education	High school graduate or less (ref.)	−	−	−	−	−	−	−	−	−	−	−	−	−
	College graduate	0.037	1.571	0.116	0.020	0.964	0.335	0.005	0.240	0.810	0.004	0.224	0.823	2.042
	More than college graduate	0.037	1.552	0.121	0.026	1.264	0.206	0.006	0.295	0.768	0.007	0.348	0.728	2.045
Household Income	3000 < (ref.)	−	−	−	−	−	−	−	−	−	−	−	−	−
	3000 ≤ & < 6000	0.061	2.991	0.003 **	0.019	1.096	0.273	0.001	0.036	0.971	−0.001	−0.045	0.964	1.484
	≥ 6000	0.061	3.015	0.003 **	0.032	1.798	0.072	0.009	0.524	0.600	0.006	0.337	0.736	1.510
Psychological factors	Personal knowledge				−0.192	−11.420	<0.001	−0.176	−10.764	<0.001	−0.174	−10.519	<0.001	1.383
	Controllability				−0.007	−0.456	0.649	−0.009	−0.613	0.540	−0.009	−0.601	0.548	1.079
	Dreadfulness				0.362	21.379	<0.001	0.331	19.879	<0.001	0.331	19.866	<0.001	1.407
	Risk known to science				0.025	1.429	0.153	0.028	1.620	0.105	0.029	1.700	0.089	1.500
	Familiarity				−0.096	−5.650	<0.001	−0.089	−5.387	<0.001	−0.089	−5.400	<0.001	1.391
Perceived level of exposures to Electromagnetic fields	Low (ref.)				−	−	−	−	−	−	−	−	−	−
	Middle (6–7)							0.116	6.306	<0.001	0.113	6.139	<0.001	1.716
	High (8–10)							0.256	13.327	<0.001	0.253	13.094	<0.001	1.893
Level of knowledge regarding Electromagnetic fields	Low (ref.)							−	−	−	−	−	−	−
	Middle (4–6)										−0.010	−0.628	0.530	1.332
	High (7–13)										0.037	2.253	0.024 *	1.372
R2 (△R2) *F*		0.049 21.676(0.000)			0.297 109.605(0.000)			0.333 112.503(0.000)			0.335(0.286) 100.004(0.000)			

Durbin-Watson 1.989; * *p*-value < 0.05, ** *p*-value < 0.01, Tolerance 0.1 ≥; no variables are affected by multicollinearity. *t*: t statistic is the coefficient divided by its standard error and this means the degree to which there is a linear relationship (relationship) between the independent and dependent variables. Ref.: Reference Group is the group that is the criterion for each group in regression analysis. R2: Coefficient of determination is the proportion of the variance for a dependent variable that’s explained by an independent variable or variables in a regression model. F: the ratio of the mean regression sum of squares divided by the mean error sum of squares
